# High-quality permanent draft genome sequence of *Bradyrhizobium* sp. Tv2a.2, a microsymbiont of *Tachigali versicolor* discovered in Barro Colorado Island of Panama

**DOI:** 10.1186/s40793-015-0006-0

**Published:** 2015-05-17

**Authors:** Rui Tian, Matthew Parker, Rekha Seshadri, TBK Reddy, Victor Markowitz, Natalia Ivanova, Amrita Pati, Tanja Woyke, Mohammed N Baeshen, Nabih A Baeshen, Nikos Kyrpides, Wayne Reeve

**Affiliations:** 1 Centre for Rhizobium Studies, Murdoch University, Murdoch, Australia; 2 Binghamton University, State University of New York, New York, USA; 3 DOE Joint Genome Institute, Walnut Creek, California, USA; 4 Biological Data Management and Technology Center, Lawrence Berkeley National Laboratory, Berkeley, California, USA; 5 Department of Biological Sciences, Faculty of Science, King Abdulaziz University, Jeddah, Saudi Arabia; 6 Center of Nanotechnology, King Abdulaziz University, Jeddah, Saudi Arabia; 7 Department of Biological Sciences, Faculty of Science, Jeddah University, Jeddah, Saudi Arabia

**Keywords:** Root-nodule bacteria, Nitrogen fixation, Symbiosis, Alphaproteobacteria, GEBA-RNB

## Abstract

*Bradyr*hizobiumsp. Tv2a.2 is an aerobic, motile, Gram-negative, non-spore-forming rod that was isolated from an effective nitrogen-fixing root nodule of *Tachigali versicolor* collected in Barro Colorado Island of Panama. Here we describe the features of *Bradyr*hizobiumsp. Tv2a.2, together with high-quality permanent draft genome sequence information and annotation. The 8,496,279 bp high-quality draft genome is arranged in 87 scaffolds of 87 contigs, contains 8,109 protein-coding genes and 72 RNA-only encoding genes. This rhizobial genome was sequenced as part of the DOE Joint Genome Institute 2010 Genomic Encyclopedia for Bacteria and Archaea-Root Nodule Bacteria (GEBA-RNB) project.

## Introduction

Legumes engage in nitrogen-fixation symbioses with bacterial partners from at least 13 genera of *Proteobacteria *[1]-[4]. Despite the high extent of phylogenetic diversity of root nodule bacteria, the very broad distribution of one particular genus (*Bradyrhizobium *) across host legume clades suggests that bacteria in this genus may have been the first legume symbionts [5]. *Bradyrhizobium * interacts with the widest diversity of legume clades (at least 24 of ca. 33 nodule-forming legume tribes; [6]) and is associated with nodulating groups that represent early branching lineages [7] in all three legume subfamilies [8],[9]. Analysis of basal *Bradyrhizobium * lineages that are associated with early-diverging legume groups may thus shed light on the origins of this symbiosis.

Here we report the genome sequence of one such organism, *Bradyrhizobium * strain Tv2a.2. Strain Tv2a.2 was sampled in 1997 from the tree Tachigali versicolor  on Barro Colorado Island, Panama, a biological preserve with an old-growth moist tropical forest [10]. *Tachigali* is one of just a handful of nodule-forming genera in the legume Subfamily Caesalpinioideae [11], which is comprised of the earliest branching lineages in the legume family [7]. Tachigali versicolor  is a large canopy tree with an unusual monocarpic life history, in which trees grow for decades without flowering. They produce just a single crop of seeds, and then die [12].

Strain Tv2a.2 is a typical representative of the nodule symbionts that are associated with *Tachigali* in this tropical forest habitat [13], and appears to represent a unique early-diverging lineage of *Bradyrhizobium *. Phylogenetic analyses have placed Tv2a.2 somewhere near the early split in the genus between two large superclades represented by *B. diazoefficiens *USDA 110 and *B. elkanii *USDA 76. However, its exact position near the base of the *Bradyrhizobium * tree varies to some extent in different analyses, depending on the loci, the strains included, and the method of tree analysis [5],[13]. For example, a Bayesian analysis of 16S rRNA sequences from the type strains of 21 *Bradyrhizobium * species and strain ORS278 placed Tv2a.2 as the earliest diverging *Bradyrhizobium * lineage [14].

Here we provide an analysis of the complete genome sequence of Tv2a.2, one of the rhizobial genomes sequenced as part of the DOE Joint Genome Institute 2010 Genomic Encyclopedia for Bacteria and Archaea-Root Nodule Bacteria (GEBA-RNB) project proposal [15], whose properties should help to clarify early events in the diversification of the genus *Bradyrhizobium * as a whole.

## Organism information

### Classification and features

**Figure 1:**
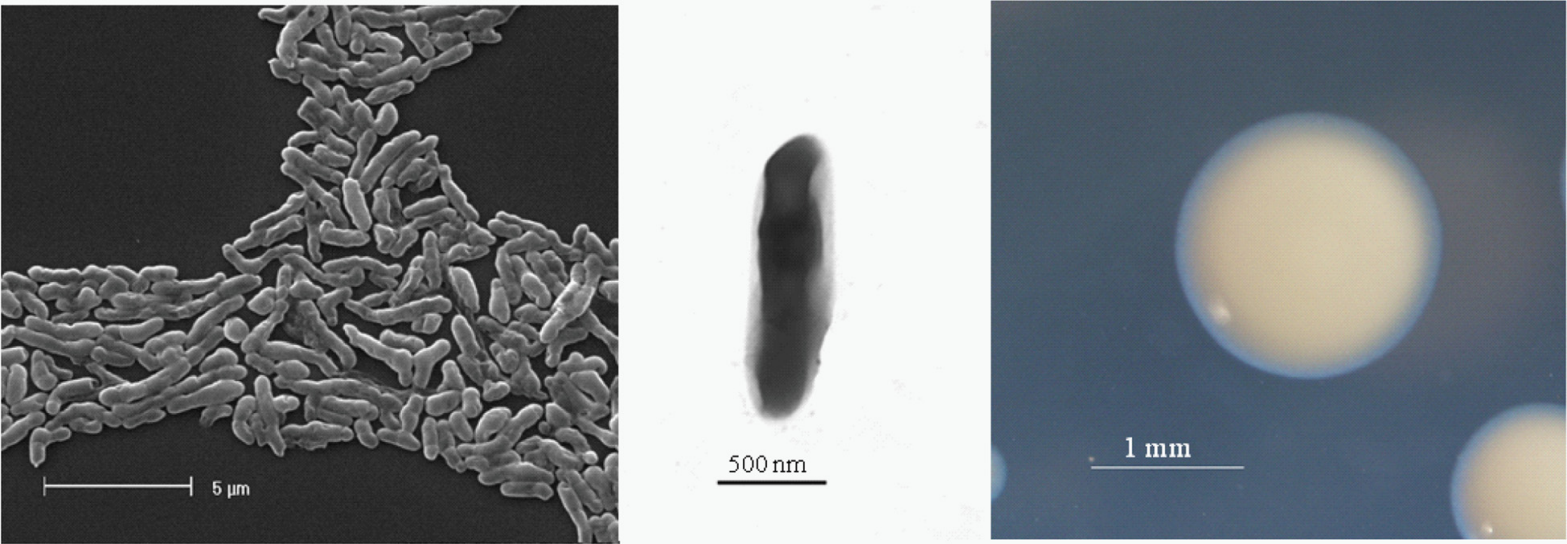
Images of *Bradyrhizobium* sp. Tv2a.2 using scanning (Left) and transmission (Center) electron microscopy as well as light microscopy to visualize colony morphology on solid media (Right).

*Bradyrhizobium * sp. Tv2a.2 is a motile, non-sporulating, non-encapsulated, Gram-negative strain in the order *Rhizobiales * of the class *Alphaproteobacteria *. The rod shaped form (Figure 1 Left, Center) has dimensions of approximately 0.5 μm in width and 1.5-2.0 μm in length. It is relatively slow growing, forming colonies after 6–7 days when grown on half strength Lupin Agar (½LA) [16], tryptone-yeast extract agar (TY) [17] or a modified yeast-mannitol agar (YMA) [18] at 28°C. Colonies on ½LA are opaque, slightly domed and moderately mucoid with smooth margins (Figure 1 Right).

**Figure 2:**
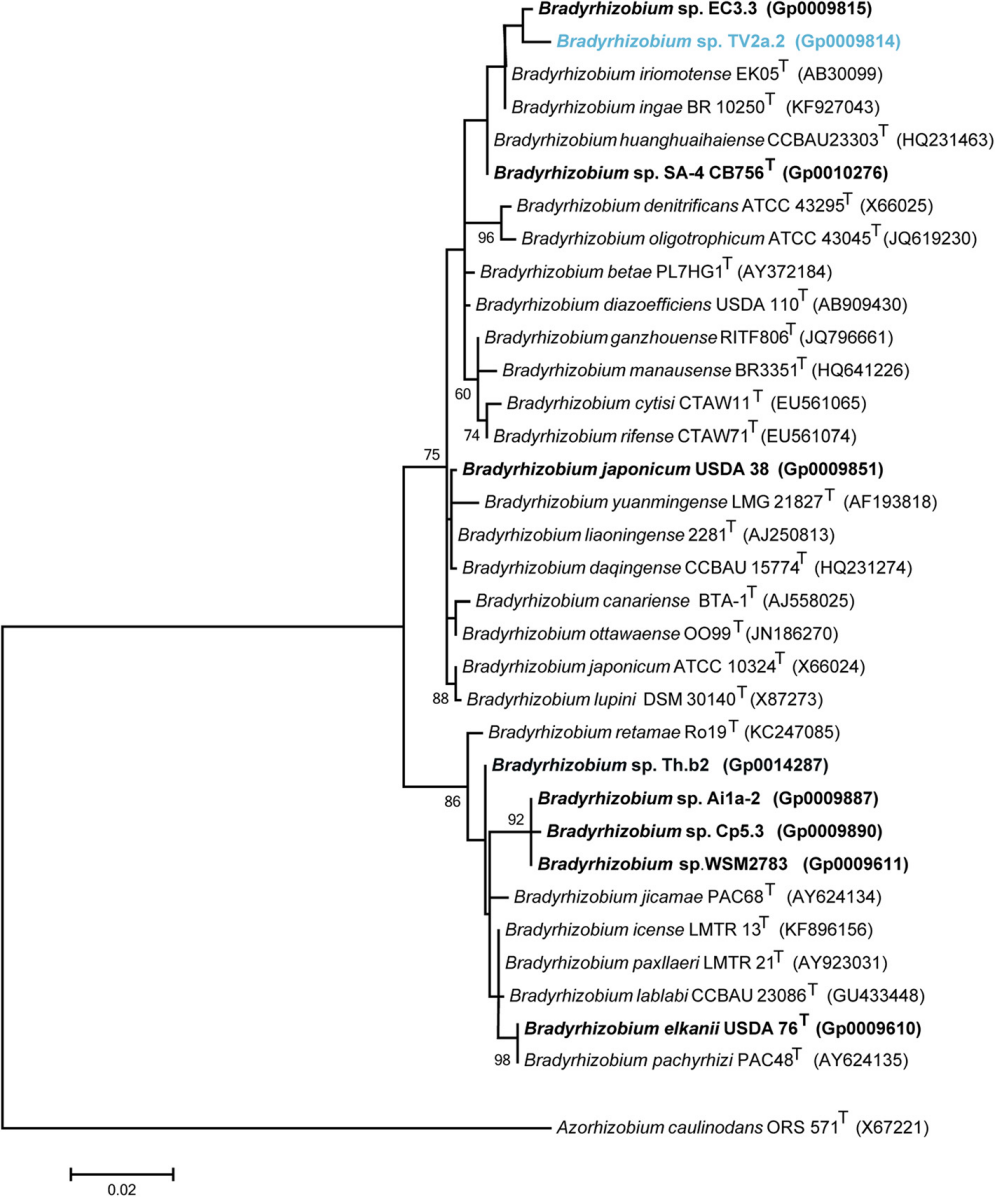
Phylogenetic tree highlighting the position of *Bradyrhizobium* sp. Tv2a.2 (shown in blue print) relative to other type and non-type strains in the *Bradyrhizobium* genus using a 1,310 bp intragenic sequence of the 16S rRNA gene. *Azorhizobium caulinodans* ORS 571 ^T^ sequence was used as an outgroup. All sites were informative and there were no gap-containing sites. Phylogenetic analyses were performed using MEGA, version 5.05 [41]. The tree was built using the maximum likelihood method with the General Time Reversible model. Bootstrap analysis with 500 replicates was performed to assess the support of the clusters. Type strains are indicated with a superscript T. Strains with a genome sequencing project registered in GOLD [22] have the GOLD ID mentioned after the strain number and are represented in bold, otherwise the NCBI accession number is provided.

Figure 2 shows the phylogenetic relationship of *Bradyrhizobium * sp. Tv2a.2 in a 16S rRNA gene sequence based tree. This strain is phylogenetically the most related to *Bradyrhizobium*sp. EC3.3 based on a 16S rRNA gene sequence identity of 99.31% as determined using BLAST analysis [19]. Tv2a.2 is also related to the type strains *Bradyrhizobium ingae * BR 10250 ^T^ and *Bradyrhizobium iriomotense * EK05 ^T^ with 16S rRNA gene sequence identities of 99.16 % and 99.08%, respectively, based on results from the EzTaxon-e server [20],[21].

Minimum Information about the Genome Sequence (MIGS) of Tv2a.2 is provided in Table 1 and Additional file 1: Table S1.

**Table 1:** **Classification and general features of *Bradyrhizobium* sp. Tv2a.2 in accordance with the MIGS recommendations**[42]**published by the Genome Standards Consortium**[43]

**MIGS ID**	**Property**	**Term**	**Evidence code**
	Classification	Domain *Bacteria*	TAS [44]
		Phylum *Proteobacteria*	TAS [45],[46]
		Class *Alphaproteobacteria*	TAS [46],[47]
		Order *Rhizobiales*	TAS [48]
		Family *Bradyrhizobiaceae*	TAS [49]
		Genus *Bradyrhizobium*	TAS [50]
		Species *Bradyrhizobium* sp.	IDA
	Gram stain	Negative	IDA
	Cell shape	Rod	IDA
	Motility	Motile	IDA
	Sporulation	Non-sporulating	NAS
	Temperature range	Unknown	NAS
	Optimum temperature	28°C	NAS
	pH range; Optimum	Unknown	
	Carbon source	Varied	NAS
	Energy source	Chemoorganotroph	NAS
MIGS-6	Habitat	Soil, root nodule, host	TAS [10]
MIGS-6.3	Salinity	Non-halophile	NAS
MIGS-22	Oxygen requirement	Aerobic	NAS
MIGS-15	Biotic relationship	Free living, symbiotic	TAS [10]
MIGS-14	Pathogenicity	Non-pathogenic	NAS
	Biosafety level	1	TAS [51]
	Isolation	Root nodule of *Tachigali versicolor*	TAS [10]
MIGS-4	Geographic location	Barro Colorado Island, Panama	TAS [10]
MIGS-5	Sample collection	1997	IDA
MIGS-4.1	Latitude	9.1663	IDA
MIGS-4.2	Longitude	- 79.8248	IDA
MIGS-4.3	Depth	5 cm	IDA
MIGS-4.4	Altitude	28 m	IDA

Evidence codes – IDA: Inferred from Direct Assay; TAS: Traceable Author Statement (i.e., a direct report exists in the literature); NAS: Non-traceable Author Statement (i.e., not directly observed for the living, isolated sample, but based on a generally accepted property for the species, or anecdotal evidence). Evidence codes are from the Gene Ontology project [52],[53].

### Symbiotaxonomy

*Bradyrhizobium * strain Tv2a.2 was isolated from nodules of Tachigali versicolor  found in a tropical forest on Barro Colorado Island, Panama [10]. Due to the highly erratic pattern of seed production from this host, no seeds of this legume were available to authenticate the symbiotic proficiency of strain Tv2a.2. Nodulation and nitrogen fixation was therefore tested on two promiscuous legumes (Vigna unguiculata *,*Macroptilium atropurpureum ) and revealed that nodules could only develop on *M. atropurpureum*. Acetylene reduction assays also showed that these nodules lacked nitrogenase activity [13]. A further indication that Tv2a.2 may be relatively host-specific is the fact that extensive sampling of other legume hosts in Panama (and elsewhere in the Neotropics) have never recovered strains belonging to the Tv2a.2 lineage from any legume taxa other than *T. versicolor*[9].

## Genome sequencing and annotation information

### Genome project history

This organism was selected for sequencing on the basis of its environmental and agricultural relevance to issues in global carbon cycling, alternative energy production, and biogeochemical importance, and is part of the Genomic Encyclopedia of Bacteria and Archaea, Root Nodulating Bacteria (GEBA-RNB) project at the U.S. Department of Energy, Joint Genome Institute (JGI). The genome project is deposited in the Genomes OnLine Database [22] and a high-quality permanent draft genome sequence in IMG [23]. Sequencing, finishing and annotation were performed by the JGI using state of the art sequencing technology [24]. A summary of the project information is shown in Table 2.

**Table 2:** Project information

**MIGS ID**	**Property**	**Term**
MIGS-31	Finishing quality	High-quality permanent draft
MIGS-28	Libraries used	Illumina Standard PE
MIGS-29	Sequencing platforms	Illumina HiSeq2000
MIGS-31.2	Fold coverage	109.04×
MIGS-30	Assemblers	Velvet version 1.1.04; Allpaths-LG version r39750
MIGS-32	Gene calling method	Prodigal 1.4
	Locus Tag	A3AI
	GenBank ID	AXAI00000000
	GenBank Date of Release	September 30, 2013
	GOLD ID	Gp0009814 [54]
	BIOPROJECT	165315
MIGS-13	Source Material Identifier	Tv2a.2
	Project relevance	Symbiotic N _2_ fixation, agriculture

### Growth conditions and genomic DNA preparation

*Bradyrhizobium * sp. Tv2a.2 was cultured to mid logarithmic phase in 60 ml of TY rich media on a gyratory shaker at 28°C [25]. DNA was isolated from the cells using a CTAB (Cetyl trimethyl ammonium bromide) bacterial genomic DNA isolation method [26].

### Genome sequencing and assembly

The draft genome of *Bradyrhizobium * sp. Tv2a.2 was generated at the DOE Joint Genome Institute (JGI) using the Illumina technology [27]. An Illumina standard shotgun library was constructed and sequenced using the Illumina HiSeq 2000 platform which generated 8,336,316 reads totaling 1250.45 Mbp. All general aspects of library construction and sequencing were performed at the JGI and details can be found on the JGI website [28]. All raw Illumina sequence data was passed through DUK, a filtering program developed at JGI, which removes known Illumina sequencing and library preparation artifacts (Mingkun L, Copeland A, Han J, Unpublished). Following steps were then performed for assembly: (1) filtered Illumina reads were assembled using Velvet (version 1.1.04) [29], (2) 1–3 Kbp simulated paired end reads were created from Velvet contigs using wgsim [30], (3) Illumina reads were assembled with simulated read pairs using Allpaths–LG (version r39750) [31]. Parameters for the assembly steps were 1) velveth: −-v --s 51 --e 71 --i 2 --t 1 --f “-shortPaired -fastq $FASTQ” --o “-ins_length 250 -min_contig_lgth 500” for Velvet and 2) wgsim: −e 0–1 76–2 76 -r 0 -R 0 -X 0. The final draft assembly contained 87 contigs in 87 scaffolds. The total size of the genome is 8.5 Mb with an average of 109.04x coverage of the genome.

### Genome annotation

Genes were identified using Prodigal [32], as part of the DOE-JGI genome annotation pipeline [33],[34]. The predicted CDSs were translated and used to search the National Center for Biotechnology Information non-redundant database, UniProt, TIGRFam, Pfam, KEGG, COG, and InterPro databases. The tRNAScanSE tool [35] was used to find tRNA genes, whereas ribosomal RNA genes were found by searches against models of the ribosomal RNA genes built from SILVA [36]. Other non–coding RNAs such as the RNA components of the protein secretion complex and the RNase P were identified by searching the genome for the corresponding Rfam profiles using INFERNAL [37]. Additional gene prediction analysis and manual functional annotation was performed within the Integrated Microbial Genomes-Expert Review (IMG-ER) system [38] developed by the Joint Genome Institute, Walnut Creek, CA, USA.

## Genome properties

The genome is 8,496,279 nucleotides with 62.20% GC content (Table 3) and comprised of 87 scaffolds. From a total of 8,181 genes, 8,109 were protein encoding and 72 RNA only encoding genes. The majority of genes (72.94%) were assigned a putative function whilst the remaining genes were annotated as hypothetical. The distribution of genes into COGs functional categories is presented in Table 4.

**Table 3:** Genome statistics for *Bradyrhizobium* sp. Tv2a.2

**Attribute**	**Value**	**% of total**
Genome size (bp)	8,496,279	100.00
DNA coding (bp)	7,163,193	84.31
DNA G + C (bp)	5,284,500	62.20
DNA scaffolds	87	100
Total genes	8,181	100.00
Protein coding genes	8,109	99.12
RNA genes	72	0.88
Pseudo genes	7	0.09
Genes in internal clusters	665	8.13
Genes with function prediction	5,967	72.94
Genes assigned to COGs	4,871	59.54
Genes with Pfam domains	6,080	74.32
Genes with signal peptides	866	10.59
Genes with transmembrane helices	1,836	22.44
CRISPR repeats	0	0.00

**Table 4:** Number of genes associated with the general COG functional categories

**Code**	**Value**	**% of total (5,458)**	**COG category**
J	184	3.37	Translation, ribosomal structure and biogenesis
A	0	0.00	RNA processing and modification
K	378	6.93	Transcription
L	142	2.60	Replication, recombination and repair
B	2	0.04	Chromatin structure and dynamics
D	29	0.53	Cell cycle control, cell division, chromosome partitioning
V	99	1.81	Defense mechanisms
T	222	4.07	Signal transduction mechanisms
M	256	4.69	Cell wall/membrane/envelope biogenesis
N	62	1.14	Cell motility
U	105	1.92	Intracellular trafficking, secretion, and vesicular transport
O	195	3.57	Posttranslational modification, protein turnover, chaperones
C	426	7.81	Energy production and conversion
G	343	6.28	Carbohydrate transport and metabolism
E	625	11.45	Amino acid transport and metabolism
F	82	1.50	Nucleotide transport and metabolism
H	204	3.74	Coenzyme transport and metabolism
I	346	6.34	Lipid transport and metabolism
P	281	5.15	Inorganic ion transport and metabolism
Q	245	4.49	Secondary metabolite biosynthesis, transport and catabolism
R	711	13.03	General function prediction only
S	521	9.55	Function unknown
-	3,310	40.46	Not in COGS

## Conclusions

*Bradyrhizobium * sp. Tv2a.2 was collected in 1997 from a nodule of the tree Tachigali versicolor  on Barro Colorado Island, Panama. Based on 16S rRNA gene analyses, Tv2a.2 is phylogenetically the most closely related to *Bradyrhizobium * sp. EC3.3 (a strain isolated from a nodule of Erythrina costaricensis  collected from Barro Colorado Island, Panama) and to the type strains *Bradyrhizobium ingae * BR 10250 ^T^ and *Bradyrhizobium iriomotense * EK05 ^T^ isolated from Inga laurina (Sw.) Willd. growing in the Cerrado Amazon region, State of Roraima, Brazil [39] and from *Entada koshunensis*, a legume available in Okinawa, Japan [40], respectively. Strain Tv2a.2 is one of 25 *Bradyrhizobium * genomes that were sequenced within the GEBA-RNB project [15]; of these, the Tv2a.2 genome has the fifth lowest genome size (8.5 Mbp), gene count (8,181) and Pfam percentage (74.32%) amongst these strains. The specific genome attributes of *Bradyrhizobium * sp. Tv2a.2 compared to the other *Bradyrhizobium * genomes will be important to understand the interactions required for the successful establishment of an effective symbiosis with the host Tachigali versicolor *.*

## Abbreviations

GEBA-RNB: Genomic Encyclopedia for Bacteria and Archaea-Root Nodule Bacteria

JGI: Joint Genome Institute

½LA: Half strength Lupin Agar

TY: Tryptone yeast

YMA: Yeast mannitol agar

CTAB: Cetyl trimethyl ammonium bromide

## Competing interests

The authors declare that they have no competing interests.

## Authors’ contributions

MP supplied the strain and background information for this project and the DNA to the JGI, TR performed all imaging, TR and WR drafted the paper, MNB and NAB provided financial support and all other authors were involved in sequencing the genome and/or editing the final paper. All authors read and approved the final manuscript.

## Additional file

## Supplementary Material

Additional file 1: Associated MIGS record. Click here for file
